# Limited prosocial emotions (LPE) specifier in conduct disorder and offending behavior: findings from a 10-year prospective longitudinal study of juveniles in residential care

**DOI:** 10.1186/s13034-023-00676-x

**Published:** 2023-11-28

**Authors:** Cyril Boonmann, David Bürgin, Nils Jenkel, Klaus Schmeck, Marc Schmid

**Affiliations:** 1 Department of Child and Adolescent Psychiatry, University Psychiatric Hospitals, University of Basel, Wilhelm Klein-Strasse 27, 4002 Basel, Switzerland; 2 Department of Forensic Child and Adolescent Psychiatry, University Psychiatric Hospitals, University of Basel, Basel, Switzerland; 3 LUMC Curium—Department of Child and Adolescent Psychiatry, Leiden University Medical Center, Leiden, The Netherlands

**Keywords:** Conduct disorder, LPE specifier, Reoffending, Juveniles, YPI

## Abstract

**Background:**

Since the introduction of the Diagnostic and Statistical Manual of Mental Disorders (DSM)-5, a limited prosocial emotion (LPE) specifier has been added to the conduct disorder (CD) diagnosis in addition to the age of onset specifier. It was suggested that this would identify a subgroup with severe antisocial and/or aggressive behavior with serious current and future (mental health) impairment. Research in recent years has shown that this is indeed a subgroup with severe antisocial behavior; however, mental health problems do not appear to differ from those of youth with CD without LPE. Most research to date has been cross-sectional. However, longitudinal research is urgently needed to better understand the predictive value of the LPE specifier. The aim of the current longitudinal study is to examine future offending behavior of youth with CD with compared to youth without the LPE specifier. In addition, the predictive value of the categorical LPE specifier and the dimensional LPE score will be examined beyond factors that are strongly associated with future offending (i.e., gender, age, and prior offending).

**Methods:**

Adolescents and young adults (12–25) with CD (assessed with the Schedule for Affective Disorders and Schizophrenia for School-Age Children—Present and Lifetime Version [K-SADS-PL]) with (N = 61) and without (N = 75) the LPE specifier (assessed with the Callous-Unemotional [CU] dimension of the Youth Psychopathic traits Inventory [YPI]) (in line with Jambroes et al., 2016) were compared on sociodemographic characteristics, mental health problems and offending behavior. Future (general and violent) offending was based on official conviction data.

**Results:**

Our results showed that youth with CD with and without the LPE specifier did not differ in self-reported and informant-reported mental health problems. However, youth with CD with the LPE specifier showed more offending behavior and personality pathology at baseline. In addition, the categorical LPE specifier was associated with future general offending, but not with future violent offending. The dimensional LPE score was associated with both future general and violent offending. However, after adjustment for gender, age, and prior delinquency, these associations disappeared, with the exception of the association between the dimensional LPE score and violent offending, which remained significant even after controlling for gender, age, and prior violent offending.

**Discussion:**

In conclusion, there seems to be evidence of a relationship between limited prosocial emotions and future offending behavior in youth with CD. This relationship, however, should not be overestimated, as there are other (static) factors (e.g. gender and prior offending behavior) that also have a strong influence on future (violent) offending behavior. Still, from a clinical point of view, a dynamic factor like prosocial emotional skills is a good focus for reducing the risk of future offending behavior.

**Supplementary Information:**

The online version contains supplementary material available at 10.1186/s13034-023-00676-x.

## Background

With the release of the fifth edition of the Diagnostic and Statistical Manual of Mental Disorders (DSM)-5; (American Psychiatric Association (APA), [6])), a new specifier, limited prosocial emotions (LPE), was added to the conduct disorder (CD) diagnosis. LPE is characterized by (1) lack of remorse or guilt; (2) callous-lack of empathy; (3) unconcerned about performance; and (4) shallow or deficient affect. At least two of these criteria should be present for at least 12 months across multiple relationships and settings (American Psychiatric Association (APA), [6]). The purpose of this specifier is to identify a specific subgroup of severely antisocial and aggressive youth, to provide information about current and future impairment and support treatment planning for youth within the heterogeneous group of youth with CD [22, 27]. However, research to date, particularly regarding future impairment of the LPE specifier, is still limited. Therefore, the aim of the current study is to examine the extent to which the LPE specifier has predictive value for future offending behavior in a sample of youth with CD. More knowledge in this regard is important in order to prioritize youth with CD who are most in need of targeted interventions [17].

Previous research has generally shown that youth with CD with and without LPE specifier showed little difference in psychopathology [10, 12, 14, 24, 36]. In contrast, youth with CD with the LPE specifier showed more antisocial/offending traits/behaviors than youth without the LPE specifier [12, 14, 24, 31, 36]. However, it should be noted that these are mainly cross-sectional studies. The number of longitudinal studies is still limited.

To our knowledge, only one study has examined the relationship between youth with and without LPE and future offending behavior. Recent research by Colins [11] on this relationship in detained adolescent females in Belgium, with a mean follow-up of just over 2 years (Range: 0.5 to 6 years), found no differences between the two groups. It is important to consider not only the relationship between the LPE specifier and future offending behavior (important from a clinical perspective), but also the strongest risk factors for future offending behavior, such gender, age, and prior offending behavior [8, 16, 23] (important from a more criminological perspective).

Therefore, the aim of the present study is to further examine the relationship between the LPE specifier (both categorically and dimensionally) and future offending behavior in youth with CD. In addition to the relationship between the LPE specifier and future offending behavior, we will also examine this relationship, adjusted for gender, age, and prior offending behavior. In doing so, we hope to contribute to the knowledge of the LPE specifier in youth with CD as an indicator of a specific subgroup of severely antisocial and aggressive youth at heightened risk for future impairment.

## Methods

### Procedure

Data were obtained from the longitudinal “Swiss Study for Clarification and Goal-Attainment in Youth Welfare and Juvenile Justice Institutions” (*Modellversuch Abklärung und Zielerreichung in stationären Massnahmen* [MAZ.]), which was conducted between 2007 and 2012. The aim of the MAZ. study was to examine mental health, psychosocial problems, and delinquent behavior of children, adolescents, and young adults in residential child welfare and juvenile justice institutions throughout Switzerland [35]. All institutions accredited by the Swiss Federal Ministry of Justice were invited to participate, of which 64 institutions (35%) agreed to participate (20 institutions in the French-speaking, 38 in the German-speaking, and 6 in the Italian-speaking part of Switzerland). Youth were admitted to these institutions through criminal law, civil law, or voluntary placement. Youth who had lived in the facility for more than 1 month prior to the assessment and who were able to complete the French, German, or Italian assessment instruments (sufficient language skills and IQ > 70) were asked to participate. Assessments consisted of clinical interviews conducted by trained psychologists, computerized self-report measures, and ratings by institutional social workers. Prior to participation, the youth, their legal guardians, and social caseworkers received verbal and written information about the study and were asked to provide informed consent. The study procedure was approved by the Ethics Committees for Research Involving Human Subjects of the Universities of Basel and Lausanne (Switzerland) and by the Institutional Review Board of the University of Ulm (Germany). For further details on the methodology, see Schmid et al. [35].

### Participants

For the current paper, data were obtained from 136 adolescents and young adults aged 12–25 years (*M*_age_ = 16.41; *SD* = 2.30; 80.2% < 18 years). The sample consisted of 66.2% (*n* = 90) males and 33.8% (*n* = 46) females (See Table 1). All participants were examined for mental disorders using the Schedule for Affective Disorders and Schizophrenia for School-Age Children-Present and Lifetime Version (K-SADS-PL) [26] and completed the Youth Psychopathic traits Inventory (YPI) [7], from which we derived the 15 items for the LPE specifier in accordance with Jambroes et al. [24]. In addition, participants completed the Youth Self-Report (YSR) [1] to assess internalizing and externalizing mental health problems, and were assessed for Personality Disorders (PDs) using the Structured Clinical Interview for DSM-IV-TR Axis II Personality Disorders (SCID-II) [18]. Finally, information on officially recorded convictions up to the end of 2017 was obtained from the Swiss Federal Statistical Office.

**Table 1:** Cross-sectional differences in overall and specific mental health problem within youth at baseline

	Total Sample	CD-LPE-	CD-LPE +		
	*N* (%)	*N* (%)	*N* (%)	Test-statistic	*p*
Gender (N = 136)				χ^2^ = 4.994, df = 1	*p* = 0.025 *
Boys/men	90 (66.2%)	43 (47.8%)	47 (52.2%)		
Girls/women	46 (33.8%)	32 (69.6%)	14 (30.4%)		
Jurisdiction of Placement (N = 131)				χ^2^ = 4.254, df = 2	*p* = *0.119*
Civil law	66 (50.4%)	38 (57.6%)	28 (42.4%)		
Penal law	48 (36.6%)	21 (43.8%)	27 (56.2%)		
Other	17 (13.0%)	12 (70.6%)	5 (29.4%)		
Nationality (N = 136)				χ^2^ = 1.459, df = 1	*p* = *0.227*
Swiss	111 (81.6%)	58 (52.3%)	53 (47.7%)		
Other	25 (18.4%)	17 (68.0%)	8 (32.0%)		
Swiss language regions (N = 136)				χ^2^ = 0.194, df = 2	*Fisher's p* = *1.000*
German	116 (85.3%)	64 (55.2%)	52 (44.8%)		
French	17 (12.5%)	9 (52.9%)	8 (47.1%)		
Italian	3 (2.2%)	2 (66.7%)	1 (33.3%)		
Trauma exposure (Criterion A; N = 99)				χ^2^ = 0.052, df = 1	*p* = *0.828*
No	27 (27.3%)	14 (51.9%)	13 (48.1%)		
Yes	72 (72.7%)	41 (56.9%)	31 (43.1%)		
Previous offense (N = 136)				χ^2^ = 3.175, df = 1	*p* = *0.075*
No	75 (55.2%)	47 (62.7%)	28 (37.3%)		
Yes	61 (44.9%)	28 (45.9%)	33 (54.1%)		
Previous violent offense (N = 136)				χ^2^ = 1.009, df = 1	*p* = *0.315*
No	113 (83.1%)	65 (57.5%)	48 (42.5%)		
Yes	23 (16.9%)	10 (43.5%)	13 (56.5%)		

	*M* (SD)	*M* (SD)	*M* (SD)	Test-statistic	*p*
Age (N = 136)	16.41 (2.30)	16.06 (2.08)	16.84 (2.5)	t(116.59) = − 1.95	*p* = *0.053*
LPE dim	33.9 (7.85)	30.59 (5.39)	37.97 (8.5)	t(97.30) = − 5.89	*p* < .001 ***
Sum Life Events (N = 136)	3.95 (3.02)	3.64 (3.04)	4.33 (2.97)	W = 1935.50	*p* = *0.121*
Sum previous placements (N = 135)	0.94 (1.35)	0.75 (1.16)	1.19 (1.55)	W = 1982.00	*p* = *0.202*
Sum previous offenses (N = 136)	1.85 (3.38)	1.29 (2.7)	2.52 (3.98)	W = 1816.50	*p* = 0.024 *
Sum previous violent offenses (N = 136)	0.3 (0.94)	0.33 (1.15)	0.26 (0.57)	W = 2129.00	*p* = *0.289*

*CD* Conduct Disorder, *LPE* Limited Prosocial Emotions, *M* Mean, *SD* Standard Deviation; Percentages are reported per row thus LPE- and LPE + equal 100%

### Instruments

*Schedule for Affective Disorders and Schizophrenia for School-Age Children—Present and Lifetime Version (K-SADS-PL)* [26]. The K-SADS-PL is a standardized, semi-structured clinical interview for the assessment of mental disorders in children and adolescents aged 6–18 years according to the fourth edition of the Diagnostic and Statistical Manual of Mental Disorders (DSM-IV) [4]. Individual responses are scored on a 4-point Likert scale (0 = no information available, 1 = not present, 2 = subthreshold level, 3 = threshold level). The psychometric properties of the K-SADS- PL have been found to be good [25]. For the current study, only CD diagnoses were included.

*Youth Psychopathic traits Inventory (YPI)* [7]. The YPI is a 50-item self-report questionnaire designed to assess the core personality traits of psychopathy in adolescents. The YPI was developed in accordance with a three- dimensional conceptualization of psychopathy [15]: an arrogant and deceitful interpersonal style (Grandiose-Manipulative Dimension), an inadequate affective experience (Callous-Unemotional Dimension), and an impulsive and irresponsible behavioral style (Impulsive-Irresponsible Dimension). The Grandiose-Manipulative dimension has four subscales: Dishonest Charm, Grandiosity, Lying, and Manipulation; the Callous-Unemotional dimension has three subscales: Callousness, Unemotional, and Remorselessness; the Impulsive- Irresponsible dimension has three subscales: Impulsiveness, Irresponsibility, and Thrill Seeking. Items are scored on a 4-point Likert-type scale ranging from 1 = does not apply at all to 4 = applies very well [7]. The psychometric properties of the instrument are generally good. A more detailed overview can be found in Boonmann et al. [9].

Only the CU dimension of the YPI was used to assess the LPE specifier; the subscale remorselessness (five items) was used to assess the “lack of remorse or guilt” LPE specifier criterion, the subscale callousness (five items) was used to assess the “callous–lack of empathy” LPE specifier criterion, and the subscale unemotional (five items) was used to assess the “shallow or deficient affect” LPE specifier criterion. The “unconcerned about performance” LPE specifier criterion cannot be assessed with the YPI, meaning that only three of the four LPE specifier criteria can be assessed with this instrument. Importantly, previous research has suggested that this criterion was not critical to test the utility of the specifier, and that the YPI can be used as an indicator of the LPE specifier [12]. If at least one item on a subscale was rated “applies very well”, the LPE specifier criterion was met. If at least two LPE specifier criteria were present, the youth was assigned to the LPE group (consistent with the methodological preceding of [24].

Next to the categorical classification of the LPE specifier, we build an LPE dimensional score summing up all items related to the dimensions of LPE, resulting in a score ranging from 15 to 60 (This score is in fact the same as the CU score).

*Youth Self-Report (YSR)* [1] / *Child Behavior Checklist (CBLC)* [2]. The YSR is a self-report questionnaire and the CBCL a third-party questionnaire both designed to assess internalizing and externalizing mental health problems. The questionnaires list approximately 120 behavioral and emotional difficulties commonly found in children and adolescents. Items are rated on a 3-point Likert scale (0 = does not apply to, 1 = somewhat or sometimes applies, 2 = very true or often applies). The YSR/CBCL provide three broadband scales: total problems (TOT), internalizing problems (INT), externalizing problems (EXT). The psychometric properties of the instruments were found to be good [3].

*Structured Clinical Interview for DSM-IV-TR Axis II Personality Disorders (SCID-II)* [18]. The SCID-II is a semi-structured interview designed to assess DSM-IV and DSM-IV-TR PD diagnoses (i.e., paranoid, schizoid, schizotypal, histrionic, borderline, antisocial, narcissistic, avoidant, dependent, obsessive–compulsive, depressive, and passive-aggressive PDs). The interview consists of 134 items rated on a 3-point Likert scale (1 = absent, 2 = subthreshold, and 3 = threshold). Categorical diagnoses are provided according to the specific diagnostic thresholds of the PDs.

*Conviction data* Conviction data (both juvenile and adult conviction data) were obtained from the Swiss Federal Statistical Office until the end of 2017, up to 10 years after the initial assessment of the study. In accordance with the publications of the Swiss Federal Statistical Office, we assessed convictions for the two most serious types of offenses (felonies, misdemeanors), excluding the most minor category of offenses (contraventions). Violent crimes were classified according to the definitions used by the Federal Statistical Office and included all crimes involving actual or threatened harm to persons, such as all forms of assault, robbery, or coercion.

### Data analytic plan

First, we present descriptive statistics for various sociodemographic characteristics, placement-specific variables, and prior delinquency for the total sample, as well as for those with and without the LPE specifier. Differences were examined using χ^2^ tests for categorical variables and t-tests and Wilcoxon-tests for dimensional variables. Wilcoxon tests were used as nonparametric alternative when assumptions for t-tests were violated. Second, we tested for differences in self-reported and professional caregiver-reported psychopathology (total, internalizing, and externalizing) and in the prevalence of PDs between participants with CD with and without the LPE specifier using t- or χ^2^ tests. Third, we used hierarchical logistic regression analyses to model general and violent offending (binary), including first the categorical or the dimensional LPE specifier/score as a predictor, then age and gender, and finally prior offending as covariates. Fourth, we fitted hierarchical negative binomial regression analyses to model the number of total and violent offenses committed, including first the categorical or the dimensional LPE specifier/score as a predictor, then additionally age and gender, and finally also prior offences as covariates. Fifth, we used Cox proportional hazards regression to model the time to subsequent general and violent offending, again including first the categorical or the dimensional LPE specifier/score as a predictor, then age and gender, and finally prior offences as covariates. The time scale of the Cox model represented the time in years from the first measurement point to the first general or violent offense or to the last measurement point in which no offense was committed (right censoring).

The statistical software used was R (Version 4.2.2) via RStudio (Version 2022.12.0, Boston, MA, USA). Descriptive analyses and model performance were analyzed using the “easystats” ecosystem for R [28–30]. Regression models were based on complete case analyses, as no missing data were identified for these study variables of interest was apparent. For sociodemographic and mental health data, with missing data, we used complete case analyses assuming Missingness at Random and report the exact number of cases included for each of these analyses. All p-values were two-tailed, and p-values < 0.05 were marked as statistically significant, exact p-values for all tests are reported in the tables.

## Results

### Sociodemographic characteristics

In total, our sample included 136 youth, all of whom were diagnosed with CD (see Table 1 for a summary of the sociodemographic characteristics). Approximately one-third of the sample were females (33.8%) and two-third were males (66.2%). The mean age of participants was 16.41 years (SD = 2.30, Range = [12; 25], 80.2% under the age of 18 years). At the time of the assessment, all participants were placed in a residential child welfare or juvenile justice institutions in Switzerland. Approximately half of the sample were placed under civil law (50.4%), one third under penal law (36.4%), and the remainder for to other reasons (13.0%). The vast majority were of Swiss nationality (81.6%). The sample is characterized by cumulative life events and a high prevalence of traumatic events. 44.9% of the sample had committed a previous offense before the assessment and 16.9% had committed a previous violent offense (see Table 1).

Participants were divided into two CD groups, those with and those without the LPE specifier; 75 participants (55.1%) did not meet the criteria for the LPE specifier, whereas 61 participants (44.9%) met the criteria. These groups differed significantly on several sociodemographic characteristics: the CD group with the LPE specifier was more likely to be male, trended to be older, and to have committed more prior offenses than the CD group without the LPE specifier. However, the groups did not differ in terms of jurisdiction of placement, nationality, Swiss language region, their exposure to life events and trauma, and number of prior placements. Unsurprisingly, those with the LPE specifier scored higher on this dimensional LPE score (see Table 1).

### LPE and mental health problems

Participants with and without the LPE specifier did not differ in their overall levels of self-reported general, internalizing, and externalizing psychopathology, nor in their levels of caregiver-reported psychopathology (see Table 2). However, significant differences were found in the prevalence of PD diagnoses, with participants with the LPE specifier having significantly higher rates of PDs than participants without the LPE specifier (41% vs. 18.1%). The largest difference was found for Cluster B PDs (including Borderline, Histrionic, Narcissistic, and Antisocial PDs). Significant differences were also found for Cluster A PDs (including Paranoid, Schizotypal, and Schizoid PDs), but not for Cluster C PDs (including Anxious-Avoidant, Dependent, and Obsessive–Compulsive PDs) (for a summary and all test statistics, see Table 2).

**Table 2:** Cross-sectional differences in overall and specific mental health problem within youth with conduct disorder with and without LPE specifier at baseline

	CD-LPE-	CD-LPE +		
	*M* (SD)	*M* (SD)	Test-statistic	*p*
Self-reported mental health problems (N = 133; ASEBA)				
General psychopathology	62.88 (10.35)	63.25 (10.22)	*t(125.12)* = *− 0.21*	*p* = *0.834*
Internalizing problems	58.5 (11.34)	58.07 (11.41)	*t(124.18)* = *0.22*	*p* = *0.828*
Externalizing problems	63.3 (9.47)	64.92 (11.18)	*t(113.77)* = *-0.89*	*p* = *0.377*
Professional caregiver-reported mental health problems (N = 134; ASEBA)				
General psychopathology	66.84 (7.72)	65.59 (6.79)	t(129.72) = 0.99	*p* = *0.325*
Internalizing problems	61.95 (9.22)	61.64 (8.58)	t(127.85) = 0.19	*p* = *0.846*
Externalizing problems	67.28 (8.82)	66.19 (7.68)	t(129.94) = 0.77	*p* = *0.445*

	*N* (%)	*N* (%)	Test-statistic	*p*
Any personality disorders (PD; SKID-II; N = 133)	13 (18.1%)	25 (41%)	*χ*^*2*^ = *7.420, df* = *1*	*p* = 0.006 **
Cluster A PD	2 (2.7%)	12 (19.7%)	*χ*^*2*^ = *8.774, df* = *1*	*Fisher’s **p* < 0.001 ***
Cluster B PD	8 (10.7%)	22 (36.1%)	*χ*^*2*^ = *11.188, df* = *1*	*p* < 0.001 ***
Cluster C PD	1 (1.3%)	5 (8.2%)	*χ*^*2*^ = *2.306, df* = *1*	*Fisher’s p* = *0.089*

*ASEBA* Achenbach System of Empirically Based Assessment; Cluster A = paranoid, schizotypal, and schizoid PDs; Cluster B = Borderline, Histrionic, Narcissistic, Antisocial PD; Insecure, Dependent and Obsessive–Compulsive PDs. Percentages are reported per column thus those with and without PD equal 100%

### LPE and future offending

A total of 59 participants (43.4%) were convicted for at least one offense during the follow-up period; 52.5% of participants with the LPE specifier and 36% of the participants without the LPE specifier. Regarding violent offences, 23 participants committed at least one violent offense during the follow-up period (16.9%); 18% of the participants with the LPE specifier and 16% of the participants without the LPE specifier. Using logistic regression analysis (estimated by Maximum Likelihood [ML]), the categorical LPE specifier trended to be associated with general offending (p = 0.055), but not with violent offending (see Tables 3 and 4). The dimensional LPE score, however, was significantly associated with both general and violent offending. Including gender and age in the models attenuated the effect of the LPE specifier/LPE score on general and violent offending. The dimensional LPE score remained significantly related to violent offending, but no longer to general offending. Gender was associated with both general and violent offending, while older age tended to reduce the odds of violent offending, but was not associated with general offending (see Tables 3 and 4 for full model specifications). Including previous offences in all these models, showed previous offences to significantly increase the odds of later offending, significant for general offences, but not for violent offences.

**Table 3:** Hierarchical logistic regression analyses for general offending over the follow-up period

Predictors	Model 1a			Model 1b			Model 1c		
	*OR*	*CI*	*p*	*OR*	*CI*	*p*	*OR*	*CI*	*p*
LPE cat	1.96	0.99 – 3.94	0.055	1.57	0.75 – 3.31	0.234	1.45	0.65 – 3.23	0.363
Gender (W)				0.21	0.08 – 0.48	** < 0.001**	0.27	0.10 – 0.65	**0.005**
Age				0.99	0.85 – 1.16	0.884	0.88	0.73 – 1.05	0.145
Prev. Offenses							5.33	2.32 – 12.83	** < 0.001**
Observations	136			136			136		
R^2^ Tjur	0.027			0.128			0.229		

	Model 1d			Model 1e			Model 1f		
Predictors	*OR*	*CI*	*p*	*OR*	*CI*	*p*	*CI*	*CI*	*p*
LPE dim	1.47	1.04 – 2.13	**0.036**	1.20	0.82 – 1.78	0.343	1.21	0.80 – 1.87	0.378
Gender (W)				0.22	0.09 – 0.51	**0.001**	0.28	0.11 – 0.71	**0.008**
Age				1.00	0.86 – 1.17	0.989	0.88	0.74 – 1.05	0.162
Prev. Offenses							5.43	2.37 – 13.06	** < 0.001**
Observations	136			136			136		
R^2^ Tjur	0.034			0.125			0.231		

*OR* Odds Ratio, *CI* 95% Confidence Interval. All dimensional predictors are standardized

**Table 4:** Hierarchical logistic regression analyses predicting violent offending over the follow-up period

Predictors	Model 2a			Model 2b			Model 2c		
	*OR*	*CI*	*p*	*OR*	*CI*	*p*	*OR*	*CI*	*p*
LPE cat	1.16	0.46 – 2.85	0.753	1.07	0.40 – 2.84	0.889	1.02	0.38 – 2.74	0.970
Gender (W)				0.14	0.02 – 0.50	**0.010**	0.15	0.02 – 0.56	**0.014**
Age				0.82	0.65 – 1.01	0.074	0.77	0.59 – 0.96	**0.030**
Prev. viol. Offenses							2.72	0.76 – 9.52	0.116
Observations	136			136			136		
R^2^ Tjur	0.001			0.086			0.101		

Predictors	Model 2d			Model 2e			Model 2f		
	*OR*	*CI*	*p*	*OR*	*CI*	*p*	*OR*	*CI*	*p*
LPE dim	1.74	1.13 – 2.74	**0.013**	1.64	1.02 – 2.71	**0.045**	1.58	0.97 – 2.63	0.070
Gender (W)				0.18	0.03 – 0.69	**0.029**	0.19	0.03 – 0.75	**0.037**
Age				0.79	0.61 – 0.98	**0.043**	0.74	0.57 – 0.94	**0.022**
Prev. viol. Offenses							2.36	0.63 – 8.52	0.189
Observations	136			136			136		
R^2^ Tjur	0.045			0.120			0.130		

*OR* Odds Ratio, *CI* 95% Confidence Interval. All dimensional predictors are standardized

In addition, we also conducted hierarchical negative binomial regression analyses (estimated using ML) with the number of general or violent offenses as outcome measure and generally found comparable patterns of estimates (see Additional file 1: Tables S1, 2). Interestingly, the dimensional LPE score remained significantly predictive of the number of both future offenses in general and future violent offenses even after controlling for covariates (i.e., gender, age, and number of prior violent offenses).

**Fig. 1:**
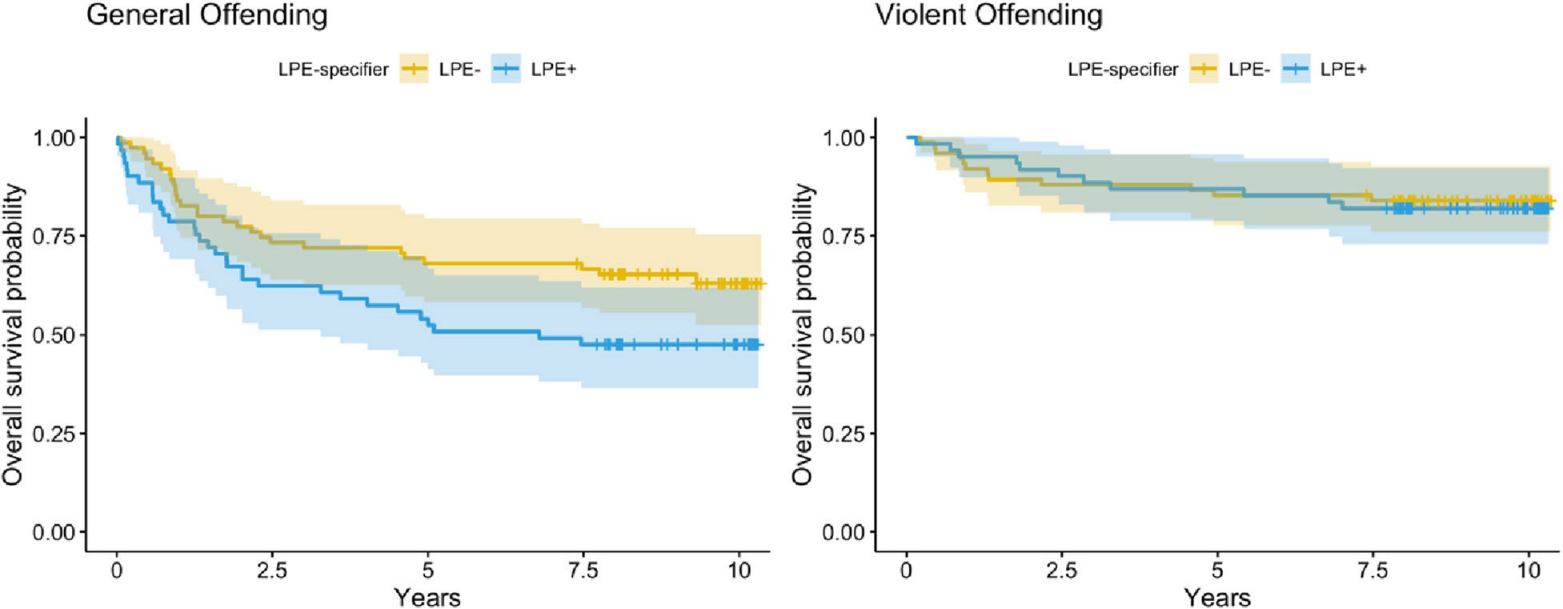
Survival plots time in years to a subsequent general offense (left) or violent offense (right) by the categorical LPE specifier. “Survivors” are participants who did not commit a general offense during the follow-up period. Error bars are 95% Confidence Intervals

**Fig. 2:**
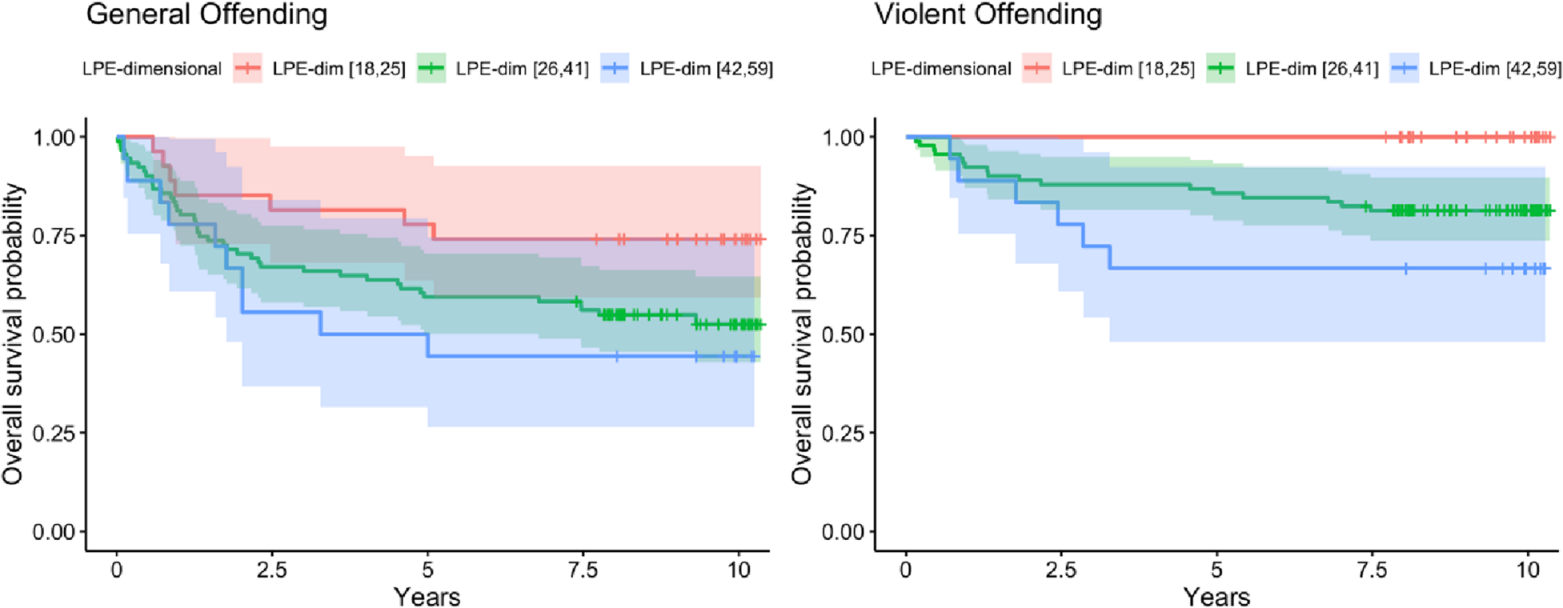
Survival plots time in years to a subsequent general offense (left) or violent offense (right) by LPE dimensional groups cut-points are ± one standard deviation from the mean. “Survivors” are participants who did not commit a general offense during the follow-up period. Error bars are 95% Confidence Intervals

The mean follow-up period for subsequent offending was 9.33 years (SD = 0.94, range = 7.4–10.4) after initial assessment. Participants who committed an offense (N = 59) committed the offense after on average 2.21 years (SD = 2.25, range = 0.01–9.31 years) after the initial assessment. Participants who committed a violent offense (N = 23) committed the offense after on average 2.6 years (SD = 2.33, range = 0.15–7.47) after the initial assessment. Unadjusted Cox proportional hazard regression analyses showed that the categorical LPE specifier and the dimensional LPE score, both significantly increased the hazard for general offending over the study period (see Table 5 and Figs. 1, 2). However, adjustment for age and gender attenuated the effect, which was not significant anymore. Gender however was significantly related to general offending over the course of the study, with men being at higher risk. Including prior offending into these models further decreased the LPE estimates, prior offending significantly increased the hazard of general offending after study assessments. In additional models, the categorical LPE specifier did not significantly increase the hazard for violent offending, but the dimensional LPE score did (and remained significant even after controlling for covariates (i.e., gender, age, and prior violent offending) (see Table 6 and Figs. 1, 2). Gender was again significantly linked to violent offending over the course of the study, with men being at higher risk (see Tables 5 and 6; gender findings displayed in Additional file 1: Figures S1, 2).

**Table 5:** Hierarchical cox regression analyses predicting the time towards general offending over the follow-up period

Predictors	Model 3a			Model 3b			Model 3c		
	*HR*	*CI*	*p*	*HR*	*CI*	*p*	*HR*	*CI*	*p*
LPE cat	1.68	1.01 – 2.80	**0.048**	1.41	0.84 – 2.39	0.2	1.30	0.76 – 2.22	0.3
Gender (W)				0.29	0.14 – 0.61	** < 0.001**	0.40	0.19 – 0.84	**0.016**
Age				0.99	0.89 – 1.09	0.8	0.90	0.81 – 1.01	0.067
Prev. Offending							3.56	1.94 – 6.53	** < 0.001**
Observations	136			136			136		
R^2^ Nagelkerke	0.029			0.126			0.235		

Predictors	Model 3d			Model 3e			Model 3f		
	*HR*	*CI*	*p*	*HR*	*CI*	*p*	*HR*	*CI*	*p*
LPE dim	1.31	1.03 – 1.65	**0.026**	1.16	0.90 – 1.48	0.2	1.13	0.89, 1.43	0.3
Gender (W)				0.30	0.15 – 0.63	** < 0.001**	0.41	0.19, 0.87	**0.020**
Age				0.99	0.90 – 1.10	> 0.9	0.91	0.81, 1.01	0.079
Prev. Offending							3.58	1.96, 6.53	** < 0.001**
Observations	136			136			136		
R^2^ Nagelkerke	0.034			0.123			0.235		

*HR* Hazard Ratio, *CI* 95% Confindence Interval. All dimensional predictors are standardized

**Table 6:** Hierarchical cox regression analyses predicting the time towards violent offending over the follow-up period

Predictors	Model 4a			Model 4b			Model 4c		
	*HR*	*CI*	*p*	*HR*	*CI*	*p*	*HR*	*CI*	*p*
LPE cat	1.11	0.49 – 2.52	0.8	1.03	0.45 – 2.39	> 0.9	0.97	0.42 – 2.27	> 0.9
Gender (W)				0.16	0.04 – 0.67	**0.013**	0.17	0.04, 0.76	**0.020**
Age				0.85	0.70 – 1.02	0.082	0.79	0.64, 0.98	**0.028**
Prev. viol. Offending							2.65	0.92, 7.65	0.072
Observations	136			136			136		
R^2^ Nagelkerke	0.001			0.111			0.135		

Predictors	Model 4d			Model 4e			Model 4f		
	*HR*	*CI*	*p*	*HR*	*CI*	*p*	*HR*	*CI*	*p*
LPE dim	1.60	1.12 – 2.28	**0.009**	1.55	1.05 – 2.30	**0.029**	1.57	1.03, 2.39	**0.038**
Gender (W)				0.21	0.05 – 0.90	**0.036**	0.22	0.05, 0.98	**0.047**
Age				0.80	0.66 – 0.99	**0.038**	0.85	0.61, 1.18	0.3
Prev. viol. Offending							1.81	0.56, 5.90	0.3
Observations	136			136			136		
R^2^ Nagelkerke	0.055			0.147			0.160		

*HR* Hazard Ratio, *CI* 95% Confindence Interval. All dimensional predictors are standardized

Due to the wide age range at baseline (12–25 years), we repeated sensitivity analyses for all models described above including only participants aged 12–18 years at baseline (N = 109, 80.2% of the total sample) (see Additional file 1: Tables S3 to 8). Overall, the patterns of findings with respect to the categorical LPE specifier and the dimensional LPE score are comparable to those of the total sample.

## Discussion

The main aim of the present study was to examine the unadjusted as well as the adjusted relationship between the LPE specifier (both categorical and dimensional) and future offending behavior in youth with CD. We found that the categorical LPE specifier was associated with future general offending, but not with future violent offending, and that the dimensional LPE score was associated with both future general and violent offending. However, after adjustment for gender, age, and prior delinquency, these relationships disappeared, with the exception of the association between the dimensional LPE score and violent offending, which tended to be or remained significant even after controlling for gender, age, and prior violent offending. Furthermore, gender emerged as the most consistent significant predictor (for both general and violent offending), whereas previous general offending significantly increased the odds and hazards for subsequent general offending over the follow-up period.

Our results showed similarities but also important differences between a categorical and a dimensional LPE specifier approach. Most notably, future violent offending behavior was related to the dimensional LPE score (even after correcting for important other predictors of future offending behavior), but not to the categorical LPE specifier. Because the LPE specifier in this study was based on the CU dimension of the YPI (consistent with [24], the dimensional LPE score should actually be referred to as a CU trait score. Based on our findings, it appears that dimensional CU traits are associated with future violent offending, whereas this does not appear to be the case for the categorical LPE specifier. Obviously, more research is needed, but it does raise the question of whether the categorical LPE specifier might be better replaced by a more dimensional (CU trait) approach. In line with current discussions in the field [13, 20, 32–34], one might even suggest using the multidimensional concept of psychopathy (Grandiose-Manipulative, Callous-Unemotional, and Impulsive-Irresponsible dimensions), instead of just the CU dimension. This needs to be further investigated in future research comparing the relationship between the LPE specifier, CU traits, and psychopathic traits and future (violent) offending behavior.

In addition, we would like to highlight two other points from the cross-sectional comparison of youth with CD with and without the LPE specifier. First, consistent with previous research, we generally found no differences in mental health problems between the CD groups with and without the LPE specifier [10, 12, 14, 24, 36], and found that the CD group with the LPE specifier exhibited more prior offenses than the CD group without the LPE specifier [12, 14, 31, 36]. However, we found that the CD group with the LPE specifier had more PDs, particularly Cluster A and Cluster B PDs, than the CD group without the LPE specifier. Although the current study, as well as previous studies, generally found no differences in mental health problems between youth with CD with and without the LPE specifier, this finding is nevertheless in line with expectations, as limited social skills and callous-unemotional traits are characteristics of Cluster A and Cluster B PDs (especially the antisocial personality disorder [ASPD]; American Psychiatric Association (APA), [5]; American Psychiatric Association (APA), [6]). Second, no differences were found between the two groups in terms of prior violent offending behavior. Interestingly, Colins and Andershed [12] found significant differences between youth with CD with and without the LPE specifier (based on the YPI and the Inventory of Callous-Unemotional Traits [ICU]; [19] for nonviolent offending, but not for violent offending. However, when the LPE specifier was based on the Antisocial Process Screening Device (APSD; [21], they found no significant differences between youth with CD with and without the LPE specifier for both nonviolent and violent offending. Van Damme et al. [36] found similar results using the APSD to establish the LPE specifier. In addition, the low base rate of violent offending may also have contributed to the non-significant difference between the two groups. If the effect size of the difference between the two groups is small, a larger sample is needed to detect this significant difference. Research with larger samples is therefore recommended.

## Limitations

The present study must be viewed in the light of some limitations. First, the LPE specifier was measured using the CU dimension of the YPI. Although previous research has shown that the YPI can be used for this purpose [12], it only takes into account three of the four characteristics of the specifier (the “unconcerned about performance” LPE specifier criterion cannot be assessed with the YPI). This could potentially underestimate the number of youth with the LPE specifier. In addition, the YPI does not take into account the time criterion (of at least 12 months). Therefore, there may be a more specific LPE subgroup within our CD sample with the LPE specifier. Furthermore, CD was based on a multi-informant, semi-structured clinical interview, whereas the LPE specifier was based on a self-report instrument. This may also have influenced the results.

Second, in addition to the LPE specifier, the CD diagnosis includes a second specifier, the age of onset specifier, which distinguishes between childhood onset (CD symptoms before age 10) and adolescent onset (no CD symptoms before age 10) CD. Unfortunately, this information was not sufficiently valid collected in our study, which prevented us from including this specifier. Interesting questions for future research include the overlap between the childhood onset specifier and the LPE specifier in youth with CD, the incremental value of the LPE specifier over the age of onset specifier for future (violent) offending behavior, and the interaction effect between the LPE specifier and the age of onset specifier for future (violent) offending behavior.

Third, our sample consisted of a heterogeneous group with both child welfare and juvenile justice youth. It should be noted, however, that all of the youth included had a CD diagnosis. In addition, it is well known that there are youth who are known to both systems, known as crossover youth. The extent to which the heterogeneity may have affected our findings could be further explored in larger/cleaner samples.

Finally, we only included criminal convictions. Hence, we did not include crimes that did not result in a conviction (e.g., due to insufficient evidence or because the case was not reported to the police) and thus may have underreported the number of offenses. Studies using self-report or police data may therefore paint a different picture. Further research, including other forms of offending behavior registration, may therefore provide a more complete picture of the (future) offending behavior of youth with CD with and without the LPE specifier.

## Implications

Based on our findings, there appears to be evidence that limited prosocial emotions/CU traits are associated with later general and violent offending behavior. However, the predictive value of the categorical LPE specifier for later offending behavior is limited and is itself nullified when important static risk factors for later offending behavior (such as gender, and prior offending) are included. Interestingly, the dimensional LPE specifier (or CU trait score) was associated with future violent offending, even when controlling for other important predictors of future offending. Therefore, it seems important to consider this dynamic risk factor (i.e., LPE/CU traits) in the screening/assessment of youth with CD and in the treatment of youth with CD with the LPE specifier and/or high levels of CU traits. However, in order to better understand the relationship between the LPE specifier/CU traits and future (violent) offending behavior, it is important to further examine the interaction, also including other important risk factors, such as the age of onset specifier, as mentioned earlier, but also other factors, such as attachment, trauma history, comorbidity, parenting style, and peer influence.

## Conclusion

In conclusion, our study shows that there is evidence of a relationship between limited prosocial emotions/CU traits and future (violent) offending behavior in youth with CD, although this relationship should not be overestimated, as there are other static factors, such as gender and prior offending behavior, that also have a strong influence on future offending behavior. However, from a clinical perspective, social-emotional skills are a good focus for reducing the risk of future (violent) offending behavior. However, further research with better operationalizations of the LPE specifier, in larger samples, focusing also on other risk factors for future (violent) offending, is still needed to better understand this relationship.

## Supplementary Information

**Additional file 1.** Supplementary material: LPE specifier and offending behavior

## Data Availability

The data sets generated and/or analyzed in the current study are not publicly available because they are property of the Federal Ministry of Justice, but are available from the corresponding author upon reasonable request.
