# Supplementary material for: Limited prosocial emotions (LPE) specifier in conduct disorder and offending behavior: findings from a 10-year prospective longitudinal study of juveniles in residential care
Source: Child Adolesc Psychiatry Ment Health. 2023 Nov 28;17:132. doi: 10.1186/s13034-023-00676-x (PMC10685542; doi:10.1186/s13034-023-00676-x)
Supplement: Supplementary file 1 — Additional file 1. Supplementary material: LPE specifier and offending behavior [file 13034_2023_676_MOESM1_ESM.pdf]

Supplementary Material: LPE specifier and Offending Behavior

Supplementary Table 1 Hierarchical negative-binomial regression analyses predicting number of *general* offenses.

	**Number of General Offenses**			**Number of General Offenses**			**Number of General Offenses**		
Predictors	*IRR*	*CI*	*p*	*IRR*	*CI*	*p*	*IRR*	*CI*	*p*
LPE+-cat.	1.92	1.04 – 3.60	**0.039**	1.69	0.93 – 3.08	0.076	1.60	0.88 – 2.91	0.107
Gender (W)				0.14	0.06 – 0.29	**<0.001**	0.15	0.07 – 0.33	**<0.001**
Age				0.90	0.79 – 1.03	0.095	0.85	0.72 – 0.99	**0.027**
Number prev. Offenses							1.09	0.98 – 1.23	0.088
Observations	136			136			136		
R^2 Nagelkerke	0.053			0.348			0.371		

	**Number of General Offenses**			**Number of General Offenses**			**Number of General Offenses**		
Predictors	*IRR*	*CI*	*p*	*IRR*	*CI*	*p*	*IRR*	*CI*	*p*
LPE dim.	1.70	1.25 – 2.37	**<0.001**	1.38	1.05 – 1.85	**0.025**	1.38	1.06 – 1.84	**0.023**
Gender (W)				0.16	0.07 – 0.34	**<0.001**	0.19	0.08 – 0.40	**<0.001**
Age				0.93	0.82 – 1.05	0.217	0.86	0.74 – 1.00	**0.043**
Number prev. Offenses							1.10	0.99 – 1.24	0.055
Observations	136			136			136		
R^2 Nagelkerke	0.143			0.371			0.402		

Supplementary Table 2 Hierarchical negative-binomial regression analyses predicting number of *violent* offenses.

	Number of Violent Offenses			Number of Violent Offenses			Number of Violent Offenses		
Predictors	*IRR*	*CI*	*p*	*IRR*	*CI*	*p*	*IRR*	*CI*	*p*
LPE cat	1.04	0.42 – 2.57	0.940	0.94	0.38 – 2.30	0.889	0.92	0.37 – 2.28	0.851
Gender (W)				0.11	0.02 – 0.41	**0.004**	0.11	0.02 – 0.43	**0.005**
Age				0.83	0.66 – 1.01	0.058	0.81	0.63 – 1.03	0.077
Number prev. Viol Offenses							1.03	0.86 – 1.21	0.752
Observations	136			136			136		
R^2 Nagelkerke	0.000			0.237			0.239		

	Number of Violent Offenses			Number of Violent Offenses			Number of Violent Offenses		
Predictors	*IRR*	*CI*	*p*	*IRR*	*CI*	*p*	*IRR*	*CI*	*p*
LPE dim.	1.75	1.15 – 2.80	**0.007**	1.56	1.04 – 2.40	**0.033**	1.56	1.03 – 2.41	**0.034**
Gender (W)				0.15	0.02 – 0.56	**0.015**	0.15	0.02 – 0.58	**0.016**
Age				0.80	0.64 – 0.98	**0.034**	0.80	0.63 – 1.01	0.066
Number prev. Viol Offenses							1.00	0.84 – 1.18	0.964
Observations	136			136			136		
R^2 Nagelkerke	0.115			0.298			0.298		

Supplementary Table 3 Hierarchical logistic regression analyses predicting general offending over the follow-up period for the subsample between 12 and 18.

	**General Offenses**			**General Offenses**			**General Offenses**		
Predictors	*OR*	*CI*	*p*	*OR*	*CI*	*p*	*OR*	*CI*	*p*
LPE cat	2.05	0.95 – 4.51	0.071	1.57	0.65 – 3.75	0.310	1.82	0.68 – 4.98	0.233
Gender (W)				0.16	0.06 – 0.40	**<0.001**	0.17	0.06 – 0.46	**0.001**
Age				1.43	1.05 – 1.97	**0.026**	1.40	1.01 – 2.01	0.052
Prev. Offenses							8.56	3.27 – 24.76	**<0.001**
Observations	109			109			109		
R^2 Tjur	0.030			0.197			0.358		
	**General Offenses**			**General Offenses**			**General Offenses**		
Predictors	*OR*	*CU*	*p*	*OR*	*CI*	*p*	*OR*	*CI*	*p*
LPE dim.	1.57	1.06 – 2.41	**0.029**	1.17	0.74 – 1.87	0.510	1.19	0.71 – 2.04	0.526
Gender (W)				0.17	0.06 – 0.43	**<0.001**	0.17	0.05 – 0.49	**0.002**
Age				1.42	1.05 – 1.98	**0.027**	1.40	1.00 – 2.01	0.055
Prev. Offenses							8.18	3.17 – 23.21	**<0.001**
Observations	109			109			109		
R^2 Tjur	0.046			0.192			0.351		

Note. OR = Odds Ratio, CI = 95% Confidence Interval.

Supplementary Table 4 Hierarchical logistic regression analyses predicting violent offending over the follow-up period for the subsample between 12 and 18.

	**Violent Offenses**			**Violent Offenses**			**Violent Offenses**		
Predictors	*OR*	*CI*	*p*	*OR*	*CI*	*p*	*OR*	*CI*	*p*
LPE cat	1.83	0.70 – 4.86	0.215	1.51	0.54 – 4.27	0.429	1.41	0.49 – 4.06	0.520
Gender (W)				0.13	0.02 – 0.49	**0.009**	0.14	0.02 – 0.55	**0.013**
Age				0.96	0.68 – 1.36	0.795	0.89	0.62 – 1.29	0.548
Prev. viol. Offenses							2.73	0.66 – 10.87	0.155
Observations	109			109			109		
R^2 Tjur	0.014			0.099			0.114		

	**Violent Offenses**			**Violent Offenses**			**Violent Offenses**		
Predictors	*OR*	*CI*	*p*	*OR*	*CI*	*p*	*OR*	*CI*	*p*
LPE dim.	2.17	1.34 – 3.71	**0.003**	1.91	1.13 – 3.45	**0.021**	1.85	1.08 – 3.40	**0.033**
Gender (W)				0.18	0.03 – 0.74	**0.034**	0.20	0.03 – 0.80	**0.044**
Age				0.88	0.61 – 1.27	0.495	0.83	0.56 – 1.22	0.351
Prev. viol. Offenses							2.37	0.52 – 10.14	0.245
Observations	109			109			109		
R^2 Tjur	0.093			0.147			0.158		

Note. OR = Odds Ratio, CI = 95% Confidence Interval.

Supplementary Table 5 Hierarchical negative-binomial regression analyses predicting number of *general* offenses over the follow-up period for the subsample between 12 and 18.

	**Number of General Offenses**			**Number of General Offenses**			**Number of General Offenses**		
Predictors	*IRR*	*CI*	*p*	*IRR*	*CI*	*p*	*IRR*	*CI*	*p*
LPE cat.	1.96	0.98 – 3.98	0.059	1.53	0.78 – 2.99	0.192	1.40	0.73 – 2.67	0.296
Gender (W)				0.13	0.06 – 0.29	**<0.001**	0.17	0.07 – 0.36	**<0.001**
Age				0.99	0.78 – 1.26	0.938	0.93	0.74 – 1.17	0.506
Number prev. Offenses							1.27	1.06 – 1.57	**<0.001**
Observations	109			109			109		
R^2 Nagelkerke	0.055			0.384			0.460		

	**Number of General Offenses**			**Number of General Offenses**			**Number of General Offenses**		
Predictors	*IRR*	*CI*	*p*	*IRR*	*CI*	*p*	*IRR*	*CI*	*p*
LPE dim.	1.77	1.27 – 2.56	**0.001**	1.38	1.01 – 1.92	**0.046**	1.35	1.00 – 1.86	0.053
Gender (W)				0.16	0.07 – 0.36	**<0.001**	0.20	0.09 – 0.44	**<0.001**
Age				1.00	0.80 – 1.24	0.979	0.93	0.75 – 1.15	0.497
Number prev. Offenses							1.25	1.06 – 1.53	**<0.001**
Observations	109			109			109		
R^2 Nagelkerke	0.165			0.414			0.492		

Supplementary Table 6 Hierarchical negative-binomial regression analyses predicting number of *violent* offenses over the follow-up period for the subsample between 12 and 18.

	**Number of Violent Offenses**			**Number of Violent Offenses**			**Number of Violent Offenses**		
Predictors	*IRR*	*CI*	*p*	*IRR*	*CI*	*p*	*IRR*	*CI*	*p*
LPE cat.	1.48	0.58 – 3.79	0.412	1.19	0.46 – 3.09	0.705	1.06	0.39 – 2.79	0.906
Gender (W)				0.11	0.02 – 0.40	**0.004**	0.12	0.02 – 0.46	**0.007**
Age				0.94	0.68 – 1.31	0.700	0.91	0.65 – 1.28	0.567
Number prev. Viol Offenses							1.12	0.92 – 1.40	0.166
Observations		109			109			109	
R^2 Nagelkerke		0.014			0.249			0.269	

	**Number of Violent Offenses**			**Number of Violent Offenses**			**Number of Violent Offenses**		
Predictors	*IRR*	*CI*	*p*	*IRR*	*CI*	*p*	*IRR*	*CI*	*p*
LPE dim.	2.00	1.31 – 3.21	**0.001**	1.76	1.14 – 2.81	**0.009**	1.70	1.11 – 2.70	**0.012**
Gender (W)				0.16	0.02 – 0.61	**0.020**	0.17	0.03 – 0.66	**0.026**
Age				0.86	0.62 – 1.17	0.326	0.84	0.61 – 1.14	0.259
Number prev. Viol Offenses							1.08	0.89 – 1.30	0.362
Observations		109			109			109	
R^2 Nagelkerke		0.193			0.345			0.355	

Supplementary Table 7 Hierarchical cox regression analyses predicting the time towards general offending over the follow-up period for the subsample between 12 and 18.

	**Time to General Offenses**			**Time to General Offenses**			**Time to General Offenses**		
Predictors	*HR*	*CI*	*p*	*HR*	*CI*	*p*	*HR*	*CI*	*p*
LPE cat.	1.75	0.99, 3.08	0.054	1.48	0.83, 2.64	0.2	1.38	0.77, 2.48	0.3
Gender (W)				0.28	0.13, 0.58	**<0.001**	0.34	0.16, 0.72	**0.005**
Age				1.24	1.00, 1.53	**0.046**	1.17	0.93, 1.47	0.2
Prev. gen. Offending							3.81	2.08, 6.99	**<0.001**
Observations	109			109			109		
R^2 Nagelkerke	0.033			0.183			0.321		

	**Time to General Offenses**			**Time to General Offenses**			**Time to General Offenses**		
Predictors	*HR*	*CI*	*p*	*HR*	*CI*	*p*	*HR*	*CI*	*p*
LPE dim.	1.37	1.06, 1.77	**0.018**	1.14	0.87, 1.48	0.3	1.08	0.83, 1.40	0.6
Gender (W)				0.28	0.13, 0.59	**<0.001**	0.33	0.15, 0.71	**0.005**
Age				1.23	0.99, 1.52	0.060	1.18	0.93, 1.48	0.2
Prev. gen. Offending							3.82	2.09, 6.98	**<0.001**
Observations	109			109			109		
R^2 Nagelkerke	0.047			0.176			0.316		

Supplementary Table 8 Hierarchical cox regression analyses LPE cat. predicting the time towards violent offending over the follow-up period for the subsample between 12 and 18.

	**Time to Violent Offenses**			**Time to Violent Offenses**			**Time to Violent Offenses**		
Predictors	*HR*	*CI*	*p*	*HR*	*CI*	*p*	*HR*	*CI*	*p*
LPE+-cat.	1.68	0.71, 3.96	0.2	1.40	0.59, 3.33	0.4	1.47	0.61, 3.52	0.4
Gender (W)				0.15	0.03, 0.65	**0.011**	0.15	0.03, 0.67	0.013
Age				0.97	0.73, 1.29	0.8	0.95	0.69, 1.29	0.7
Prev. viol. Offending							2.30	0.77, 6.86	0.13
Observations	109			109			109		
R^2 Nagelkerke	0.015			0.124			0.160		

	**Time to Violent Offenses**			**Time to Violent Offenses**			**Time to Violent Offenses**		
Predictors	*HR*	*CI*	*p*	*HR*	*CI*	*p*	*HR*	*CI*	*p*
LPE dim.	1.85	1.29, 2.65	**<0.001**	1.69	1.12, 2.54	**0.012**	1.57	1.03, 2.39	**0.038**
Gender (W)				0.21	0.05, 0.93	**0.040**	0.22	0.05, 0.98	**0.047**
Age				0.88	0.64, 1.20	0.4	0.85	0.61, 1.18	0.3
Prev. viol. Offending							1.81	0.56, 5.90	0.3
Observations	109			109			109		
R^2 Nagelkerke	0.105			0.176			0.185		

Supplementary Figures

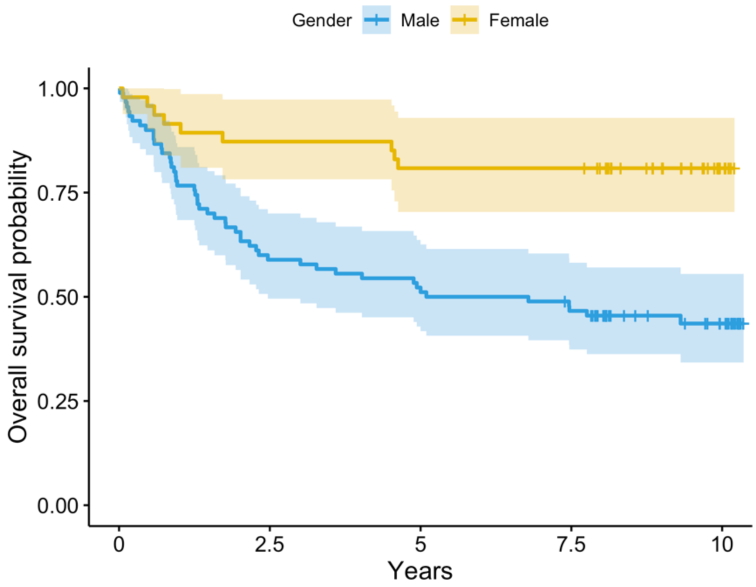

Supplementary Figure 1. Survival plot time in years to subsequent general offenses by Gender. Those who survived are participants who did not commit general offenses during the follow-up period.

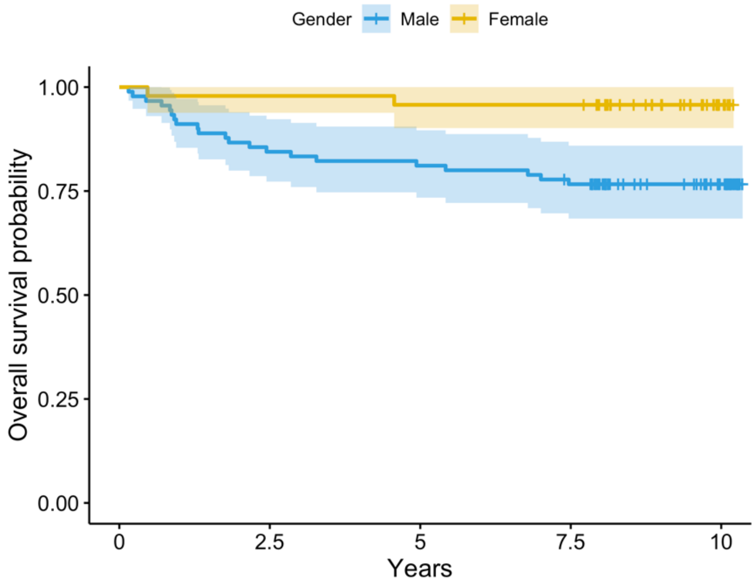

Supplementary Figure 2. Survival plot time in years to subsequent violent offenses by Gender. Those who survived are participants who did not commit general offenses during the follow-up period.
